# Effects of prilled fat supplementation in diets with varying protein levels on production performance of early lactating Nili Ravi Buffaloes

**DOI:** 10.5713/ab.23.0543

**Published:** 2024-04-26

**Authors:** Saba Anwar, Anjum Khalique, Hifzulrahman, Muhammad NaeemTahir, Burhan E Azam, Muhammad Asim Tausif, Sundas Qamar, Hina Tahir, Murtaza Ali Tipu, Muhammad Naveed ul Haque

**Affiliations:** 1 Buffalo Research Institute, Pattoki District Kasur, 53000, Pakistan; 2 Department of Animal Nutrition, University of Veterinary and Animal Sciences, Lahore 54000, Pakistan; 3 Department of Livestock Production, University of Veterinary and Animal Sciences, Lahore 54000, Pakistan; 4 Department of Livestock Management, Faculty of Veterinary and Animal Sciences, The Islamia University, Bahawalpur 63100, Pakistan; 5 Livestock Experiment Station, Bhunikey, Pattoki, District Kasur, 53000, Pakistan; 6 Department of Livestock and Dairy Development, Lahore 54000, Pakistan

**Keywords:** Buffalo, Methane, Milk Production, Protein Supplies, Rumen Inert Fat

## Abstract

**Objective:**

The objective of the current study was to find out the independent and interactive effects of prilled fat supplementation with protein on the production performance of early lactating Nili Ravi buffaloes.

**Methods:**

Sixteen early lactating buffaloes (36.75±5.79 d in milk; mean±standard error) received 4 treatments in 4×4 Latin-square design according to 2×2 factorial arrangements. The dietary treatments were: i) low protein low fat, ii) low protein high fat, iii) high protein low fat, and iv) high protein high fat. The dietary treatments contained 2 protein (8.7% and 11.7% crude protein) and fat levels (2.6% and 4.6% ether extract) on a dry matter basis.

**Results:**

The yields of milk and fat increased with increasing protein and fat independently (p≤0.05). Energy-, protein-, and fat-corrected milk yields also increased with increasing protein and fat independently (p≤0.05). Increasing dietary protein increased the protein yield by 3.75% and lactose yield by 3.15% and increasing dietary fat supplies increased the fat contents by 3.93% (p≤0.05). Milk yield and fat-corrected milk to dry matter intake ratios were increased at high protein and high fat levels (p≤0.05). Milk nitrogen efficiency was unaffected by dietary fat (p>0.10), whereas it decreased with increasing protein supplies (p≤0.05). Plasma urea nitrogen and cholesterol were increased by increasing protein and fat levels, respectively (p≤0.05). The values of predicted methane production reduced with increasing dietary protein and fat.

**Conclusion:**

It is concluded that prilled fat and protein supplies increased milk and fat yield along with increased ratios of milk yield and fat-corrected milk yields to dry matter intake. However, no interaction was observed between prilled fat and protein supplementation for production parameters, body weight, body condition score and blood metabolites. Predicted methane production decreased with increasing protein and fat levels.

## INTRODUCTION

Water buffaloes (*Bubalus bubalis*) are the second most important dairy animal and their milk is enriched with various essential nutrients such as fat, protein, and minerals. Despite the high milk protein content, the average milk yield of lactating buffalo is relatively low [1]. One of the primary reasons for the low productivity of buffalo is inadequate feeding practices. Buffaloes are generally fed with available seasonal fodder along with wheat or rice straws. These feedstuffs contain low supplies of fermentable protein and carbohydrates/energy [2] leading to suboptimal milk production.

Energy is the major limiting factor affecting the productivity of dairy animals and in the case of buffaloes, where milk fat to protein ratio is higher compared to cows, the energy requirements become even more limiting. Increasing energy supplies through cereal grains [3] or fat supplementation [4] can be effective mechanisms to improve milk production. However, there are certain limitations to overfeeding grains, as it could induce acidosis in the rumen. On the other hand, fat supplementation has the potential benefit of increasing energy intake, without the risk of acidosis [3]. Nevertheless, excessive fat supplementation, could lead to lowered fermentation of fiber in the rumen depending upon the degree of saturation [5].

The most common fats fed to dairy animals are saturated and unsaturated. The saturated fats are less digestible and affect fiber digestion in a lesser acute way than the unsaturated fats at the rumen level due to their inert nature [6]. Prilled fat is one of the rumen-inert fats, rich in palmitic acid (PA; C16:0), designed to minimize negative effects on rumen fermentation [4]. Various studies of dairy cows [6,7] and buffaloes [4] have reported increased milk production by feeding rumen inert fat.

Literature relating to the addition of fat [4] and protein [8] to the diet of buffalo for improving production performance is available but their interaction effect has not been described in buffalo as per authors’ knowledge. It would be interesting to explore the interaction of fat with protein on the milk production and composition in buffalo. Moreover, the increased milk fat to protein ratio in buffalo milk indicates that the demand of energy for milk production might be higher compared to cows [1]. Similar experiments were conducted with dairy cows [9,10] but milk yield, composition and component yields were different between cows and buffalo. Therefore, the current study aimed to explore the independent or interactive effects of increasing dietary protein and prilled fat supplies on production performance of early lactating buffaloes, with the hypothesis that high protein in combination with high fat (HF) supplies would improve the milk and milk components yield.

## MATERIALS AND METHODS

### Buffaloes

The study was conducted at the Livestock Experiment Station, Bhunikey, Buffalo Research Institute located at Pattoki, Punjab, Pakistan (31.02°N, 73.85°E, and 186 m altitude) from November, 2020 to January, 2021. The entire study was performed according to ethical rules and regulations for animal welfare approved by farm management vide letter No. 124 of dairy section LES, Bhunikey. Sixteen multiparous buffaloes in early lactation with the following details were enrolled: (mean± standard deviation) 9.52±1.41 kg/d of milk yield, 5.56%± 0.76% of milk fat, 537±72.39 kg of live body weight, and 36.75 ±5.79 days in milk (DIM). The experimental animals were individually tied in a ventilated shed and fresh water was available during the whole day.

### Experimental design, treatments, and feeding

Sixteen early lactating buffalo were blocked by their milk yield, and divided into 4 squares in 4×4 Latin square design such that within each square buffalo had similar milk yield. Then within each square, buffaloes were assigned randomly to the treatment in a 2×2 factorial arrangement with four 21 days (d) periods. The total duration of the experiment was 84 d excluding the pre-experiment period. The treatments consisted of: i) LPLF, low protein low fat; ii) LPHF, low protein high fat; iii) HPLF, high protein low fat; and iv) HPHF, high protein high fat. The low (8.7%) and high (11.7%) crude protein (CP) per kg diet dry matter (DM) levels were achieved by manipulating corn gluten (30%), canola and soybean meal in the concentrate mixture, whereas HF (4.6% ether extract [EE]/kg diet DM) levels were achieved by adding 300 g/d PA (C16:0, 86%) in top-dressed fashion in the form of prilled fat. The low fat (LF) treatment (2.6% EE/kg diet DM) had no supplementation of prilled fat. The diets were formulated using Cornell-Pen-Miner-Dairy 3.0.10 software based on Cornell Net Carbohydrate and Protein System version 5.0.2 for Holstein Friesian cows (developed at Cornell University, Ithaca, NY, USA; University of Pennsylvania, Philadelphia, PA, USA; and Miner Institute Chazy, NY, USA) on the basis of nutrient requirements established in previous studies of protein [8,11] and fat levels [4] in buffaloes. The diets were composed of 30% corn silage, 24% to 26% wheat straw, and 44% concentrate with or without 2% prilled fat, and offered in restricted amount in the form of total mixed ration once during 24 hours period at 0900 h. The details of ingredients used in the diets and their chemical composition are given in Table 1. A period of one week was provided to experimental animals as an adaptation period. During adaptation period, animals were fed combination of all four dietary treatments in homogenous form. Buffaloes were relatively similar in DIM; hence, similar lactation persistency was assumed throughout the study.

### Experimental measures, sample collections, and analysis

The samples of each feedstuff (corn silage, wheat straw and concentrates) were collected twice in each period to determine the DM (method 934.01) and composited for further laboratory analysis. After the estimation of DM content in corn silage, the quantity of silage offered was adjusted twice in a period to ensure the same delivery of DM on each day. There was no refusal of diet and feed offered (14 kg) in restricted amounts was the actual intake on a DM basis. These samples were evaluated for CP (984.13, N×6.25; Kjeldahl method), EE (method 920.39), and ash (method 942.05) by following the AOAC International official methods (2005). Analysis of neutral detergent fiber (NDF with the addition of α-amylase and sodium sulfite) and acid detergent fiber (H_2_SO_4_+CTAB) were performed by Ankom-2000 fiber analyzer (Fairport, NY, USA). Starch content of concentrate was measured according to Hodge and Hofreiter [12] and molasses sugar was measured according to Kitinoja and Awad [13]. Corn starch was determined by using a near infrared spectrophotometer (NIRS DS200; Foss, Hilleroed, Denmark). All these values were used in the CNCPS system to predict the starch and sugar values of dietary treatments. Animals were milked two times daily at 0500 and 1700 h. Milk samples from morning and evening milking were collected on alternate days in the first 2 weeks and then daily in the 3rd week of each period and were analyzed separately using an ultrasonic milk analyzer (Lactoscan S 1720; Milkotronic, Nova Zagora, Bulgaria) for fat, protein, and lactose contents. Blood samples were taken from the Jugular vein of buffaloes on the 3rd last day (18th day) of each period following [11]. Heparinized syringes were used for blood sample collection and it was immediately centrifuged at 4°C for 15 min at 2,000×g. The plasma was separated with micropipettes, aliquoted in 1 mL Eppendorf tubes, and stored at −20°C to be analyzed by using commercially available enzymatic kits (Randox Laboratories Ltd., County Antrim, UK). The concentrations of glucose (GL2623), plasma urea nitrogen (PUN; UR107), triglyceride (TR210), and cholesterol (CH201) were measured by using a biochemical analyzer (RX Monza; Randox Laboratories Ltd., UK). Buffaloes were weighed before the experiment and then at the end of every period, after morning milking and before feeding. Similarly, body condition score (BCS) was measured before and at the end of each period by following Ferguson et al [14], independently assessed by 3 individuals throughout the experiment, and a median score was used for each buffalo.

### Calculations and statistical analysis

The non-fibrous carbohydrates were determined by formula = 100–(CP+NDF+EE+ash) according to NRC [5]. Energy-corrected milk (ECM), 4% fat-corrected milk (FCM) and protein-corrected milk (PCM) were estimated using the equations mentioned previously [8]. Feed efficiency = milk yield/DMI, gross efficiency of MP = milk protein yield/MP intake and metabolic efficiency of MP = milk protein yield/(MP intake –MP for growth, maintenance and pregnancy) were calculated. Milk nitrogen efficiency (MNE) and Milk N (MkN) were calculated as: MNE = (N in milk/N intake)× 100 and Milk N = milk true protein/6.38 by using equations mentioned in Akhtar et al [8]. The marginal efficiency of fat was calculated as = fat yield/EE intake. The marginal efficiency of production parameters for NEL consumed was estimated by the formulas reported by Moallem [15]. The marginal efficiency of MY = milk yield/(NE_C_–NE_M_), the marginal efficiency of 4% FCM = 4% FCM/(NE_C_–NE_M_), and the marginal efficiency of ECM = ECM/(NE_C_–NE_M_). Net energy content (NE_C_) = (NE_L_ per kg of DM)×DMI and NE_M_ (net energy for maintenance) = body weight^0.75^×0.08×1.1. Methane production, yield and intensity were estimated using equations described by Patra [16].

Data collected on a daily basis were condensed to weekly means before the statistical analysis. Data from the 3rd week (last week) of each study period were analyzed using the MIXED procedure of SAS University Edition [17]. The statistical model included the fixed effect of protein, fat, an interaction between protein and fat, and period. Square and buffaloes nested within square were considered as random effects in the following model.


$${\text{Y}}_{ijklm}=\mu +{\text{S}}_{i}+{\text{Buff}}_{\text{j(i)}}+{\text{Per}}_{\text{k}}+{\text{PT}}_{\text{l}}+{\text{FT}}_{\text{m}}+{\left(\text{PT}\times \text{FT}\right)}_{\text{lm}}+{ɛ}_{\text{ijklm}}$$

where Y is the response variable (variable of interest), μ is the overall mean, S_i_ is the random effect of square (i = 1 to 4), Buff_j(i)_ represents the random effect of buffalo within square (i = 1 to 4), Buff_j(i)_ represents the random effect of buffalo within square (j = 1 to 4), Per_k_ represents the fixed effect of period (k = 1 to 4), PT_l_ = fixed effect of protein (l = 1 to 2), FT_m_ = fixed effect of fat (m = 1 to 2), (PT×FT)_lm_ = fixed effect of interaction of PT and FT and ɛ_ijklm_ = residual random error term. The values reported in the tables are least square means of PT×FT with standard errors of the means, and treatment differences were considered significant when p≤0.05 and tendency was set at 0.05<p≤0.10.

## RESULTS

### Milk production and composition

The DM intake was the same for all dietary treatments (14.0 kg/animal/d). The lactation responses of buffaloes during different treatments are presented in Table 2. There was no protein×fat interaction for milk yield or components (p>0.10). Increasing dietary protein supplies increased the yields of milk by 4.63% (p<0.01), fat by 5.56% (p<0.01), protein by 3.75% (p<0.01), and lactose by 3.17% (p = 0.02). Increasing dietary protein supplies increased the ECM yield by 4.71% (p<0.01), FCM yield by 5.00% (p<0.01), and PCM yield by 7.76% (p<0.01). Contents of fat, protein and lactose were not affected by increasing the levels of protein (p>0.10). The milk energy (MkE) increased by 4.35% on the high protein diet (p<0.01). The MkN increased by 3.79% with increasing protein supplies in the treatments (p = 0.01).

Increasing the levels of fat increased the milk yield by 3.19% (p = 0.03), fat yield by 6.65% (p<0.01), and fat content by 3.93% (p<0.01), whereas decreased the protein content by 0.77% (p = 0.04) and lactose content by 0.59% (p = 0.05). Neither protein yield nor lactose yield were affected by varying fat levels (p>0.10). The HF diet increased the ECM and FCM yields by 4.71% and 5.73%, respectively (p≤0.01). The MkE increased by 4.35% (p<0.01) and protein-corrected milk yield tended to increase by 5.11% (p = 0.07) with increasing fat levels, whereas MkN was not affected (p = 0.15). Body weight increased by dietary protein supplies (p = 0.03), whereas not affected by fat supplies (p = 0.32). The dietary treatments did not affect the body weight change and BCS of experimental animals (p>0.10).

### Feed and production efficiencies

Feed and production efficiencies are given in Table 3. There was no interaction between protein and fat on any efficiency (p>0.10). Increasing dietary protein supplies, enhanced the ratio of milk yield to DMI by 3.50% (p<0.01), the ratio of FCM to DMI by 5.00% (p<0.01), and the ratio of PCM to DMI by 7.19% (p<0.01). The high protein diet decreased the gross efficiency of MP by 8.23%, metabolic efficiency of MP by 13.27%, and MNE by 23.15% compared with the low protein diet (p<0.01). The MkN to MkE ratio remained unaffected (p = 0.28). The marginal efficiency of fat and marginal efficiencies of MY, FCM, and ECM were not affected by protein supplies (p>0.10).

The HF diet increased feed efficiency (ratio of milk yield to DMI) by 2.08% (p = 0.05), ratio of FCM to DMI by 5.00% (p<0.01) and tended to increase the ratio of PCM to DMI (p = 0.08) compared with the LF diet. The HF diet tended to increase the gross and metabolic efficiency (p≤0.09). The MNE was not affected by increasing dietary fat supplies (p = 0.13). The MkN to MkE ratio decreased by 2.70% with increasing dietary fat supplies (p<0.01). The marginal efficiency of fat and marginal efficiencies of MY, FCM, and ECM were decreased by increasing the fat supplies (p<0.01).

### Blood metabolites

The responses of plasma metabolites are given in Table 4. There was no interaction between protein and fat levels for blood metabolites (p>0.10). Plasma urea nitrogen was increased by 34.87% (p<0.01), whereas cholesterol, triglycerides, and glucose were unaffected by increasing dietary protein in the treatments (p>0.10). The cholesterol increased by 10.82% (p = 0.01) and glucose decreased by 5.25% (p = 0.04), whereas triglycerides did not change in response to dietary fat supplies (p = 0.65).

### Methane production

Methane production (CH_4_) decreased with increasing the protein and fat supplies (Table 5). However, no interaction was observed between protein and fat for methane production (p>0.10). Increasing the protein supplies, reduced the CH_4_ production (MJ) by 1.37%, (MCal) by 1.43%, (g/d) by 1.23%, CH_4_ yield (g/kg of DMI) by 1.71% and CH_4_ intensity (g/kg of milk yield) by 5.62% (p<0.01). Similarly, increasing the dietary fat supplies, decreased the CH_4_ production (MJ) by 0.69%, (MCal) by 0.86%, (g/d) by 0.82%, CH_4_ yield (g/kg of DMI) by 1.14% and CH_4_ intensity (g/kg of milk yield) by 3.25% (p<0.01).

## DISCUSSION

The objective of the study was to find out the independent and interactive effects of protein and prilled fat supplementation on the lactation performance of multiparous buffaloes. The prilled fat supplement used in this study was concentrated in PA (C16:0), designed to reduce negative effects on rumen fermentation [4]. The effects of increased milk yield by protein feeding are consistent with the previous study [18]. Additionally, supplementation of prilled fat, specifically PA (C16:0), was reported to improve milk and fat yield [6,7]. Different studies of protein [8,11] and fat feeding [4] were reported in dairy buffaloes but their interaction effect has not been explored in lactating buffaloes. The fixed quantity of diets was offered for three reasons; firstly, duration of period in the present study was too short to observe the effects on intake completely (normally, a period of 6 weeks is considered sufficient). Secondly, the commercial practice of farmers in our country is never to feed buffaloes *ad libitum*, especially in intensive farming. Thirdly, our focus was to observe the clear effect of the change in specific nutrient intake, and if the intake exceeded the target, it could create a problem.

### Milk yield was increased by feeding protein and prilled fat

Milk yield increased with protein supply from 8.7% to 11.7% on a DM basis. These findings are similar with previous study on buffalo [11] or cows [18]. The possible reason for increased milk yield in the present study was likely due to an increase in energy along with protein supplies. This increase may be attributed to efficient utilization of ammonia in the rumen. An important factor for increased nitrogen utilization was the carbohydrate availability [19]. The impact of increasing protein supplies on milk production was found to be greater when fed with high-energy diets [20], indicating an interaction between energy and protein for efficient nitrogen utilization. It possibly increased microbial protein synthesis in rumen which supplied most amino acids (nutrients) to small intestine [5] that ultimately improved the milk production in mammary gland. Another reason for improved milk yield was likely due to increased protein synthesis in the mammary gland because protein also serves as an osmotic agent in mammary epithelial cells along with lactose and minerals [21]. In the present study, a one-percent increase in protein supply caused about 1.4 percent increase in milk yield (0.21 kg/d/unit increase in CP) which is less than reported for Holstein cows i.e. 1.69% [22] during early to mid-lactation (1 to 150 DIM) in high protein diets (from 11.4% to 17.3% CP of diet DM) or buffaloes i.e. 1.85% [8] during mid lactation (126 to 189 DIM) in low protein diets (from 9.26% to 11.4% CP of diet DM) and greater than reported for Holstein cows i.e. 0.66% [23] during mid-lactation (120-onwards DIM) in high protein diets (from 13.5% to 16.5% CP of diet DM). Colmenero and Broderick [23] documented a quadratic trend for milk yield; the milk yield increased when CP levels were raised from 13.5% to 16.5%/kg diet DM but it decreased when the CP levels were raised above 16.5%/kg diet DM in lactating cows. The increase in non-structural carbohydrate to CP (NSC/CP) ratio improved the milk yield [24]. Contrary to our results, Naveed-ul-Haque et al [11] reported reduced milk yield by increasing the CP supplies. The possible explanation for decreased milk yield in their study was due to imbalance between energy and protein, decreased nonstructural carbohydrate to crude protein (NSC/CP) ratio, increased MP/NE_L_ ratio at isocaloric diets. The imbalance between protein and energy at rumen level may increase the bypass amounts of the undigested or partially digested nutrients to the small intestine. The digestion and metabolism processes taking place post-ruminally cannot be compared with reticulo-rumen, due to differences in mode of digestion and metabolism taking place at both places. The hind guts have limited capacity of enzyme production and inability to metabolize some of the ingredients. Thereby decreasing the amounts of metabolizable nutrients to the animals [25]. The case of imbalance is highly appreciable in situations where dietary protein is deficient or in excess and cannot meet the production requirements of microbial carbon skeleton.

The increase in milk yield in response to enhanced fat levels was consistent with findings of studies conducted on lactating buffaloes [4,26] and cows [7]. A possible explanation for the increased milk yield in the current study was due to increased energy supply provided by prilled fat supplementation. Moreover, a large proportion of the absorbed long chain fatty acid could be oxidized by the extrahepatic tissues (muscles) by fat feeding and less available for milk but other fuels like fatty acids (FA) are spared for milk synthesis [27]. Furthermore, Lohrenz et al [28] reported that bypass fat spared glucose (required for de novo FA synthesis) for more lactose synthesis, which in turn results in increased milk output without changing lactose content. However, supplemented fat had no effects on milk or its components [6,29]. The differences in the above-mentioned results were possibly due to various products (lipogenic or glucogenic) of bio hydrogenation pathways associated with different dietary fat sources i.e. more frequent incorporation of surplus energy into milk and less frequently into the milk fat or other components [6,30]. In the present study, a one-percent increase in fat supply caused about 1.59 percent increase (0.16 kg/d/unit increase in fat) in milk yield which is less than reported for buffaloes i.e. 1.85% (0.17 kg/d/unit increase in fat) [31] during early lactation (56-onwards DIM) in similar fat diets (from 3.95% to 5.21% EE of diet DM). There was no interaction between protein and prilled fat for milk yield. Our results are consistent with previous literature [9,10], which reported no interaction between fat and protein for milk yield.

### Fat, protein and lactose yields were increased by feeding protein

Protein supplies increased the milk protein and lactose yield. Increased milk protein yield was related to more milk volume [32]. This production response is in line with previous literature [8,18,23]. A linear increase in protein yield was observed by 5.3% with increasing from low to high MP supplies [18]. Increasing the MP supplies to udder enhanced the milk protein synthesis [32], mainly through enhancing milk yield by supplying α-lactalbumin for synthesis of lactose.

Milk fat yield increased by 5.52% with protein supplies. The fat yield increased due to an increase in the milk volume in the present study. The highest fat yield was observed in a high protein and HF treatment. Our results agree with the literature [8,18,23,24]. It was likely due to high metabolizable protein supplies enhanced the oxidation rate of amino acid to CO_2_ (entering into tricarboxylic acid cycle resulting in ATP synthesis) led to an increased de novo short-chain FA synthesis in the udder. Additionally, high protein supplies indirectly increase the FA supply to the udder by increasing the chylomicrons and lipoproteins production in the blood [5]. In contrast to our findings, fat yield was unaffected by protein feeding [11].

We observed no effect on fat content by increasing protein supplies in agreement with the findings of [18]. These findings contradict the results of Akhtar et al [8] who reported that fat content increased with increasing protein. This difference was possibly due to the use of mid lactating buffaloes in the study of Akhtar et al [8], whereas in our study, animals were in early lactation stage. Animals have a natural tendency to increase milk fat content in late lactation compared to early lactation, which could be a factor of lack of effect of protein feeding on milk fat in our study.

No interaction was observed between protein and prilled fat for protein yield in our study. These results are consistent with the findings of [9,10] who reported protein treatment, as well as its interaction with conjugated linoleic acid (CLA), did not affect the protein yield. It was possible that the increased protein quantity present for absorption was not enough to exhibit milk protein response with CLA feeding [10].

### Milk fat yield and content were increased by feeding prilled fat

The fat yield increased by prilled fat feeding, similar to previous studies [4,7]. The increased fat yield observed by prilled fat in the current study was due to high dietary energy content and increased preformed FA (16-C FA) concentration. Prilled fat can increase energy content of diet, probably changing the milk fat synthesis pathway from fatty acids, sparing glucose utilization in the udder, and directly producing fat from the fatty acids and glycerol to enhance the milk fat yield. This increase in milk fat yield could also be due to a greater proportion of fatty acid intake that was directly shifted to the milk fat [33]. However, contrary to our finding, Warntjes et al [34] reported that dietary fat supplementation did not change the milk fat yield.

Milk fat content was increased by prilled fat in present study, that is in agreement with the findings of [4,6]. The direct relationship between dietary, plasma and milk FA contributed to an increased milk fat in the fat supplemented group. This could be one of the reasons for the increased fat content in the current study. Another possibility for increased milk fat was that dietary fat enhanced the supply of FA to udder from the diet, which decreases de-novo fat synthesis. Improved milk fat content and yield in the current study were likely because of dietary incorporation of C16:0 into milk fat because C16:0 is preferably utilized into milk fat by the udder, compared to other FA [35] and the additional C16:0 consumed is partitioned to milk fat directly [34]. Another study by Shelke et al [36] documented that enhanced milk fat content was due to the higher availability of saturated and unsaturated FA for absorption in the intestine and then these FA were directly added to milk fat after absorption. Nichols et al [9] also reported an increase in milk fat content because fat supplies fatty acids after absorption from the intestine directly incorporated into milk fat. However, contrary to our findings, milk fat contents showed no effect in buffaloes [29] by fat feeding. This was likely due to differences in the type of feed, duration of treatment and physiological stage of the animals. Milk yield and components improved with increasing protein and fat supplies because protein quantity was sufficient to enhance the production response with the HF level in the current study.

### Body weight and body condition score

Body weight increased by the protein supplies agree with the study of Katiyar et al [26]. However, it remained unaffected by the fat supplies in the current study supported by previous studies of fat feeding [29,33]. In contrast to our results, different studies reported no effect on body weight by protein supplies [8,11,22,23]. Similarly, BCS was not affected with increasing protein and fat supplies and these findings are similar with the study of Law et al [22]. One possible reason for similar BCS in current study was likely due to high energy and amino acids provided by fat and protein feeding, that were not utilized for body fat deposition rather delivered to the udder for milk production and components. Ranjan et al [29] reported similar body weight by fat supplies because extra energy supplied through the fat feeding was not utilized for body fat deposition rather it was delivered to produce more milk fat and FCM yield.

### Milk N efficiency was decreased by feeding protein

Milk N efficiency reduced with increased protein supplies in the current study. These results are in line with previous studies in cows [18,23] or buffalo [8,11]. The increased dietary CP supply enhances the production and oxidation of amino acids in the gut epithelium, liver, and peripheral tissues including the udder. Another possible reason for decrease efficiency is linked with the metabolism in non-mammary tissues, which reduced the amino acid supply to the udder [21]. Increasing protein content of the diet increases rumen ammonia, and not all the ammonia is utilized by microbes to make microbial protein, the extra ammonia is changed to urea in the liver and appeared in the plasma pool [37]. Urea is excreted out through the urine mostly, reducing milk nitrogen efficiency. The lower N efficiency with increasing protein supplies is possibly supported by enhanced catabolism of amino acid, as indicated by the increased plasma urea N observed in the current study.

### Plasma urea N and cholesterol were increased by feeding protein and fat, respectively

Protein supplies increased the plasma urea N, these findings are in line with previous studies [8,11,23]. It was likely due to rapid degradation of dietary protein rather than microbial protein synthesis or an imbalance of fermentable energy and N available. The increased plasma urea depicts more detoxification of surplus ammonia in the liver as a result of high protein supplies in the diet. Thus, ammonia which is carried to the liver through blood is changed into urea [37]. Reduced MNE on increased protein supply in the current study supports our findings of inefficient use of nitrogen. Increasing fat supplies enhanced cholesterol and it is supported by previous study of Ranjan et al [29] due to more dietary FA uptake by the liver and the portal drained viscera. However, in the study of Singh et al [38], cholesterol remained unaffected by supplementing 2.5% bypass fat in diet.

Fat and protein supplies did not affect the triglyceride level. These results are similar to earlier studies on fat [29,38] and protein feeding [11,18]. These findings are opposite to Nichols et al [9] who reported fat feeding increased the triglyceride, but it was unaffected by protein feeding. They reported that fat feeding increased the hydrolysis of mammary triglyceride. Glucose tended to decrease by fat supplies and remained unaffected by the protein supplies in current study. Our results are supported by the findings of Law et al [22]. The glucose level was not altered significantly as the homeostatic mechanism of the body did not allow glucose to change suggested by Shelke et al [36].

### Methane production were decreased by feeding protein and prilled fat

In the present study, increasing fat and protein supplies both decreased the methane production, which is similar with the previous findings of Beauchemin et al [39]. They reported that more concentrate feeding increased the energy density of diet, decreased the amount of structural carbohydrates, increased outflow of rumen, and reduced rumen pH, decreasing CH_4_ production, yield, and intensity. In the current study, increasing protein and fat supplies increased the diet energy density which resulted in low methane emissions on high protein or fat diets.

## CONCLUSION

Under the feeding conditions of current study, dietary prilled fat and protein supplies increased milk and fat yield along with increased ratios of milk yield and FCM to DMI in early lactating buffaloes. The protein feeding increased the yields of milk fat, protein, and lactose, and decreased MNE, whereas the fat feeding increased the milk fat content and yield. However, no interaction was observed between protein and prilled fat supplementation on milk composition, milk production, blood metabolites, live body weight and BCS. Predicted methane production decreased with increasing dietary protein and fat supplies. Based on present study findings, it can be concluded that diet containing a high level of protein and fat may be used for early lactating buffaloes, as it has positive effects on production performance under restricted feeding conditions.

## Figures and Tables

**Table 1:** Ingredient and nutrient composition of dietary treatments

Items	Dietary treatments^1)^			

	LPLF	LPHF	HPLF	HPHF
Ingredient (% of DM)				
Corn silage	30.7	30.6	30.6	30.6
Wheat straw	25.5	23.6	25.4	23.5
Corn grain	12.6	12.5	10.9	10.9
Wheat brans	11.1	11.0	11.6	11.5
Molasses	4.73	4.72	3.29	3.28
Soybean hulls	8.61	8.60	2.30	2.30
Corn gluten meal (30%)	2.69	2.68	4.87	4.86
Canola meal	1.57	1.57	4.97	4.96
Soybean meal	1.57	1.57	4.96	4.95
Mineral mixture^2)^	0.40	0.40	0.40	0.40
Dicalcium phosphate	0.20	0.20	0.20	0.20
Salt (NaCl)	0.25	0.25	0.25	0.25
Urea (246%)	0.15	0.15	0.25	0.25
Prilled fat	-	2.12	-	2.12
Nutrient composition (% of DM)				
DM	55.8	55.9	56.0	56.1
CP	8.70	8.64	11.7	11.7
Ash	6.34	6.49	6.44	6.59
NDF	48.5	46.9	46.3	44.8
ADF	31.4	30.4	29.6	28.6
NFC^3)^	35.4	35.1	34.6	34.3
EE	2.57	4.65	2.63	4.70
Predicted nutritive value^4)^				
MP (g/kg of DM)	76.6	76.3	86.6	85.6
RUP (% CP)	29.6	29.4	28.6	28.4
RDP (% CP)	70.4	70.6	71.4	71.6
ME (Mcal/kg)	2.21	2.41	2.28	2.47
NEL (Mcal/kg)	1.42	1.55	1.47	1.59
Sugar (% of DM)	5.72	5.69	5.70	5.67
Starch (% of DM)	20.7	20.6	20.6	20.5
RDP:RUP^5)^	2.38	2.40	2.50	2.52

DM, dry matter; CP, crude protein; NDF, neutral detergent fiber; ADF, acid detergent fiber; NFC, non-fiber carbohydrates; EE, ether extract; MP, metabolizable protein; RUP, ruminal undegradable protein; RDP, ruminal degradable protein; ME, metabolizable energy; NE_L_, net energy for lactation.

1) LPLF, low protein low fat; LPHF, low protein high fat; HPLF, high protein low fat; HPHF, high protein high fat. low and high protein levels (8.7% and 11.7% CP). low and high fat levels (2.4 and 4.6% EE) on DM basis in diets.

2) Mineral mixture contained 0.7% DCP, 0.23% salt, 0.05% MgSO_4_, 0.007% FeSO_4_, 0.005% ZnSO_4_, 0.005% MnSO_4_, 0.0013% CuSO_4_, 0.001% CoCl, 0.005% KI. Prilled fat 300 g added to high fat treatment (2% of diet DM) while, low fat had no additional fat.

3) NFC = 100–(CP+NDF+ash+EE).

4) Predicted using CNCPS evaluation by CPM software.

5) RDP:RUP, ratio of RDP to RUP.

**Table 2:** Milk production and composition of buffaloes fed with different protein and fat levels

Items	Dietary treatments^1)^				SEM	p-value^2)^		
	
	LPLF	LPHF	HPLF	HPHF		PT	FT	PT×FT
DMI (kg/d)	13.96	13.99	13.98	14.01	0.053	0.67	0.47	1.00
Milk (kg/d)	9.86	10.1	10.2	10.6	0.385	<0.01	0.03	0.59
Yield (g/d)								
Fat	648	682	675	729	35.8	<0.01	<0.01	0.40
Protein	384	390	397	406	13.9	<0.01	0.15	0.80
Lactose	499	509	514	526	18.8	0.02	0.13	0.86
Milk composition (%)								
Fat	6.61	6.82	6.61	6.91	0.168	0.44	<0.01	0.52
Protein	3.89	3.87	3.89	3.85	0.028	0.31	0.04	0.38
Lactose	5.04	5.03	5.04	4.99	0.035	0.16	0.05	0.23
ECM^3)^ (kg/d)	14.6	15.1	15.1	16.0	0.68	<0.01	<0.01	0.46
4% FCM^4)^ (kg/d)	13.7	14.3	14.2	15.2	0.68	<0.01	0.01	0.42
3.4% PCM^5)^ (kg/d)	11.4	11.8	12.1	12.9	0.90	<0.01	0.07	0.56
MkE^6)^ (Mcal/d)	10.2	10.5	10.5	11.1	0.47	<0.01	<0.01	0.50
MkN^7)^ (g/d)	60.1	61.1	62.2	63.6	2.17	0.01	0.15	0.80
BW (kg)	523	522	531	526	26.71	0.03	0.32	0.47
BW change^8)^ (kg/d)	−0.43	−0.36	−0.04	−0.32	0.161	0.18	0.52	0.29
BCS	3.02	2.98	3.02	3.03	0.078	0.44	0.79	0.44

SEM, standard error of the mean; DMI, dry matter intake; ECM, energy corrected milk; FCM, fat corrected milk; PCM, protein corrected milk; MkE, milk energy; BW, body weight; BCS, body condition score.

1) LPLF, low protein low fat; LPHF, low protein high fat; HPLF, high protein low fat; HPHF, high protein high fat. Low and high protein levels (8.7% and 11.7% CP), low and high fat levels (2.4% and 4.6% EE) on DM basis in diets.

2) PT, main effect of protein; FT, main effect of fat; PT×FT, interaction between fat and protein effect.

3) ECM = (12.95×fat yield)+(7.65×true protein yield)+(0.327×milk yield).

4) 4% FCM = (0.4×milk yield)+[15×(fat/100)×milk yield].

5) 3.4% PCM = milk(kg)×0.294% CP.

6) MkE = 0.00929×g of fat/d + 0.00563×g of true protein/d + 0.00395×g of lactose/d.

7) Milk N = milk true protein/6.38.

8) BW change (kg/d) = body weight change.

**Table 3:** Production efficiencies of buffaloes fed with different protein and fat levels

Items	Dietary treatments^1)^				SEM	p-value^2)^		
	
	LPLF	LPHF	HPLF	HPHF		PT	FT	PT×FT
Feed efficiency^3)^	0.71	0.72	0.73	0.75	0.027	<0.01	0.05	0.59
4% FCM: DMI	0.98	1.02	1.02	1.08	0.049	<0.01	<0.01	0.44
3.4% PCM: DMI	0.82	0.85	0.87	0.92	0.064	<0.01	0.08	0.56
Gross efficiency MP^4)^	0.36	0.37	0.33	0.34	0.012	<0.01	0.08	0.66
Metabolic efficiency MP^5)^	0.56	0.57	0.48	0.50	0.029	<0.01	0.09	0.65
Milk nitrogen efficiency^6)^	30.8	31.4	23.6	24.2	0.999	<0.01	0.13	0.97
MkN: MkE^7)^ (g/Mcal)	5.93	5.80	5.91	5.72	0.109	0.28	<0.01	0.52
Marginal efficiency of fat^8)^	1.81	1.05	1.84	1.11	0.077	0.13	<0.01	0.64
Marginal efficiency for NE_L_ consumed^9)^								
Milk yield (kg/Mcal)	0.90	0.78	0.88	0.79	0.054	0.53	<0.01	0.31
4% FCM (kg/Mcal)	1.25	1.11	1.23	1.13	0.086	0.95	<0.01	0.28
ECM (kg/Mcal)	1.33	1.17	1.30	1.19	0.088	0.84	<0.01	0.30

SEM, standard error of the mean; FCM, fat corrected milk; DMI, dry matter intake; PCM, protein corrected milk; MP, metabolizable protein; MkE, milk energy; NE_L_, net energy for lactation; CP, crude protein; EE, ether extract; ECM, energy-corrected milk; NE_C_, Net energy content; NE_M_, net energy for maintenance.

1) LPLF, low protein low fat; LPHF, low protein high fat; HPLF, high protein low fat; HPHF, high protein high fat. Low and high protein levels (8.7% and 11.7% CP), low and high fat levels (2.4% and 4.6% EE) on DM basis in diets.

2) PT, main effect of protein; FT, main effect of fat; PT×FT, interaction between fat and protein effect.

3) Feed efficiency = milk yield/DMI.

4) Gross efficiency MP = milk protein yield/MP intake.

5) Metabolic efficiency MP = milk protein yield/MP intake – (MP for growth+P for maintenance+ MP for pregnancy).

6) Milk N efficiency = (N in milk/N intake)×100.

7) MkN/MkE = milk nitrogen (g/d)/milk energy.

8) Marginal efficiency of fat = fat yield/EE intake

9) Marginal efficiency of production parameters for NE_L_ consumed; milk yield/(NE_C_–NE_M_); 4% FCM/(NE_C_–NE_M_); ECM/(NE_C_–NE_M_). NE_C_ = (NE_L_ per kg of DM)×DMI; NE_M_ = BW^0.75^×0.08×1.1.

**Table 4:** Blood metabolites (mg/dL) of buffaloes fed diets with different protein and fat levels

Items	Dietary treatments^1)^				SEM	p-value^2)^		
	
	LPLF	LPHF	HPLF	HPHF		PT	FT	PT×FT
Glu	75.1	73.8	77.3	70.6	3.31	0.79	0.04	0.16
PUN	12.8	13.3	17.3	17.9	0.76	<0.01	0.38	0.89
TG	143	148	132	145	8.8	0.49	0.22	0.65
Chol	117	124	114	132	7.5	0.50	0.01	0.17

SEM, standard error of the mean; Glu, glucose; PUN, plasma urea nitrogen; TG, triglyceride; Chol, cholesterol.

1) LPLF, low protein low fat; LPHF, low protein high fat; HPLF, high protein low fat; HPHF, high protein high fat. Low and high protein levels (8.7% and 11.7% crude protein), low and high fat levels (2.4% and 4.6% ether extract) on dry matter basis in diets.

2) PT, main effect of protein; FT, main effect of fat; PT×FT, interaction between fat and protein effect.

**Table 5:** Predicted methane production (CH_4_) of buffaloes fed with different protein and fat levels

Items	Dietary treatments^1)^				SEM	p-value^2)^		
	
	LPLF	LPHF	HPLF	HPHF		PT	FT	PT×FT
CH_4_^3)^ (MJ)	14.6	14.5	14.4	14.3	0.06	<0.01	<0.01	0.98
CH_4_^4)^ (Mcal)	3.50	3.47	3.45	3.42	0.016	<0.01	<0.01	0.98
CH_4_^5)^ (g/d)	245	243	242	240	0.8	<0.01	<0.01	0.98
CH_4_^6)^ (g/kg DMI)	17.6	17.4	17.3	17.1	0.02	<0.01	<0.01	0.73
CH_4_^7)^ (g/kg Milk)	25.3	24.5	23.9	23.0^c^	0.87	<0.01	0.01	0.89

SEM, standard error of the mean; DMI, dry matter intake; NDF, neutral detergent fiber.

1) LPLF, low protein low fat; LPHF, low protein high fat; HPLF, high protein low fat; HPHF, high protein high fat. Low and high protein levels (8.7% and 11.7% crude protein), low and high fat levels (2.4% and 4.6% ether extract) on dry matter basis in diets.

2) PT, main effect of protein; FT, main effect of fat; PT×FT, interaction between fat and protein effect.

3) CH_4_, MJ = methane production in mega joule = (0.436+0.678×DMI+0.697×NDF intake).

4) CH_4_, Mcal= methane production in mega calorie = CH_4_, MJ/4.18.

5) CH_4_, g/d = methane production in gram per day = (0.671/40)×methane production in mega joule×1,000.

6) CH_4_, g/kg DMI = methane yield in gram per kg of DMI = CH_4_, g/d/DMI.

7) CH_4_, g/kg milk = methane intensity in gram per kg of milk (Patra [16]).
